# Exploring the Therapeutic Potential of *Ganoderma lucidum* in Cancer

**DOI:** 10.3390/jcm13041153

**Published:** 2024-02-18

**Authors:** Gabriella Cancemi, Santino Caserta, Sebastiano Gangemi, Giovanni Pioggia, Alessandro Allegra

**Affiliations:** 1 Hematology Unit, Department of Human Pathology in Adulthood and Childhood “Gaetano Barresi”, University of Messina, via Consolare Valeria, 98125 Messina, Italy; gabriella.cancemi@polime.it (G.C.); santino.caserta@polime.it (S.C.); 2 Allergy and Clinical Immunology Unit, Department of Clinical and Experimental Medicine, University of Messina, Via Consolare Valeria, 98125 Messina, Italy; gangemis@unime.it; 3 Institute for Biomedical Research and Innovation (IRIB), National Research Council of Italy (CNR), 98164 Messina, Italy; giovanni.pioggia@irib.cnr.it

**Keywords:** *Ganoderma lucidum*, reishi, nutraceutical, leukemia, lymphoma, hematological malignancies, immune response, apoptosis, MAP-K system

## Abstract

Triterpenoids, such as ganoderic acid, and polysaccharides, including β-D-glucans, α-D-glucans, and α-D-mannans, are the main secondary metabolites of the medicinal fungus *Ganoderma lucidum*. There is evidence of the effects of ganoderic acid in hematological malignancies, whose mechanisms involve the stimulation of immune response, the macrophage-like differentiation, the activation of MAP-K pathway, an IL3-dependent cytotoxic action, the induction of cytoprotective autophagy, and the induction of apoptosis. In fact, this compound has been tested in twenty-six different human cancer cell types and has shown an anti-proliferative activity, especially in leukemia, lymphoma, and myeloma lines. Moreover, research clarified the capability of molecules from *Ganoderma lucidum* to induce mitochondrial damage in acute promyelocytic leukemia cells, without cytotoxic effects in normal mononuclear cells. Active lipids extracted from the spores of this fungus have also been shown to induce apoptosis mediated by downregulation of P-Akt and upregulation of caspases-3, -8, and -9. Among in vivo studies, a study in BALB/c mice injected with WEHI-3 leukemic cells suggested that treatment with *Ganoderma lucidum* promotes differentiation of T- and B-cell precursors, phagocytosis by PBMCs, and NK cell activity. Our review presents data revealing the possibility of employing *Ganoderma lucidum* in hematological malignancies and incorporating it into clinical practice.

## 1. Introduction

### General Considerations on Ganoderma lucidum

*Ganoderma lucidum (G. lucidum)* is the most well-known therapeutic mushroom [1,2,3,4]. Other names for it include “lingzhi” in Chinese, “reishi” in Japanese, “yeongji” in Korean, and “ling chih” and “ling chi mushroom” in other countries; it is also called “mushroom of immortality,” “mushroom of spiritual potency,” and “spiritual plant” [5]. It belongs to the kingdom Fungi, division Basidiomycota, class Basidiomycetes, subclass Homobasidiomycetes, order Polyporales, family Ganodermataceae, genus *Ganoderma,* and species *G. lucidum* and grows on oak and plum trees, occurring in different colors and shapes [3]. Six strains of *G. lucidum* are described in Chinese medical texts, with different names depending on the colors of fruiting bodies: sekishi (red), kokushi (black), shishi (purple), seishi (blue), hakushi (white), and oushi (yellow) [6,7,8].

*G. lucidum* is a large source of pharmacologically active substances, and, because of its therapeutic benefits, it has been used for thousands of years in traditional Chinese and Japanese medicine to boost the immune system and improve health and longevity [9,10,11]. Nowadays, it is employed in the nutraceutical, dietary supplement, and cosmetic industries, and it is used as medication in the form of powder and pills [12,13,14,15]. Normally, *G. lucidum* is well tolerated; however, its intake can cause side effects such as gastrointestinal disorders, osteoarticular pain, dizziness, and skin rashes [16,17].

The numerous studies conducted have shown that it possesses antioxidant [18,19], immunomodulator [20,21], anti-allergic [22], anti-inflammatory [23,24,25], anticancer [26,27], carcinostatic [28], antiangiogenic [29,30], proapoptotic [18], cardioprotective [31], hepatoprotective [32], hypoglycemic [33], hypocholesterolemic [19], antiviral [34], anti-HIV [35], antifungal [36], antibacterial [37], and antiandrogenic activity [38]; in fact, it has been used to treat several chronic disorders, including hepatopathy, nephritis, diabetes, hypertension, arthritis, asthma, bronchitis, migraine, and sleeplessness. Moreover, it has been acknowledged particularly as alternate adjuvant therapy for cancer [11], being their bioactive compounds and extracts tested in different types of cancer such as lung cancer [39,40], prostate cancer [18,41], colon cancer [42], and breast cancer, but also in hematological malignancies like leukemia and lymphomas [23,43,44,45,46].

## 2. Search Strategy

We researched data on PubMed, with no restrictions on the date of publication, using the keywords “*Ganoderma lucidum*”, “Leukemia”, “Lymphoma”, “*Ganoderma lucidum* and leukemia”, “*Ganoderma lucidum* and lymphoma”, “Cancer”, and “Apoptosis”. Non-English language articles were excluded.

## 3. A Glance at Therapeutic Characteristics of *Ganoderma lucidum*

### Secondary Metabolites of Ganoderma lucidum

The species *G. lucidum* has been extensively studied for its secondary metabolites and the biological activities of extracts from fruiting bodies, spores, and cultured mycelia [47], containing glycoproteins, amino acids, enzymes, vitamins, polysaccharides, triterpenoids, meroterpenoids, sesquiterpenoids, steroids, alkaloids, benzopyran derivatives, and benzoic acid derivatives [48,49]. The fungus showed a quantity of 242 different proteins differentially regulated; in fact, after deeper analysis conducted using the log2fold change and the normalized peptide-spectrum match, their heatmap showed 5.74% upregulated and 94.25% downregulated proteins [50]. The composition of *G. lucidum* extracts depends on the type of solvent used; polar extracts consist mainly of polysaccharides, while apolar extracts contain triterpenes [5].

In the past 40 years, according to phytochemical reports, 431 secondary metabolites have been isolated from various species of *Ganoderma*, of which 279 have been isolated from *G. lucidum* species [17,47]. Due to their exceptional pharmacological capabilities, polysaccharides, such as fudan-yueyang-*G. lucidum* (FYGL), are regarded as the primary group of bioactive chemicals; in detail, FYGL is a proteoglycan of 2.6 × 105 Dalton as molecular weight and its moieties consist of rhamnose, arabinose, galactose, and glucose, covalently binding to the threonine and serine residues of protein moieties in fudan-yueyang-*G. lucidum* via O-type glycoside [51]. FYGL is constituted by three different fractions: the heteropolysaccharide FYGL-1 with a molecular weight of 78 kD, the proteoglycan FYGL-2 with a molecular weight of 61 kD and the highly branched FYGL-3, with a molecular weight of 100 kD [52].

Polysaccharides from *G.lucidum* have a significant impact on some diseases and, in particular, possess immunomodulatory and anticarcinogenic activities [51]. In fruiting bodies, polysaccharides make about 10 to 50 percent of the dry mass and more than 200 distinct polysaccharides have been discovered from spores, fruiting bodies, and mycelia, including β-D-glucans, α-D-glucans, α-D-mannans, and polysaccharide-protein complexes [53,54].

As for triterpenes, more than 150 ganoderic acid derivatives have been obtained from *G. lucidum* and other *Ganoderma* species, including ganoderiol, lucialdehyde, lucidenic acid, lanostanoid, ganolucidinic acids, and ganodermantriol [55]; in detail, the three lanostane triterpenoids 11*β*-hydroxy-lucidone, 11*β*-hydroxy-ganoderiol, and 24*E*-en-11-oxo-ganoderiol were isolated from fruiting bodies of the mushroom and showed an important cytotoxic activity against six mammary adenocarcinoma cell lines with IC_50_ values less than 20 μM [49]. Triterpenoids are highly oxygenated and pharmacologically active compounds that contain a carboxylic acid group commonly called ganoderic acid and are characterized by a complex structure, molecular mass, and high lipophilicity [56]. It is well known that immune response has a crucial role in the development of tumors, and certain drugs, such as cyclophosphamide, target both neoplastic cells and healthy immune cells of the patient, causing a lower rate of cancer recovery. Since it was shown that prolonged chemotherapy often leads to a depletion of T cells, scientists focused on a possible role of ganoderic acid in this field and found that in animal models of cyclophosphamide immunosuppression, it could act to improve STAT3 and TNF expression and enhance immune system response [57].

It was reported that various bioactive proteins of *G. lucidum*, including LZP-1, LZP-2, LZP-3, and LZ-8, which were extracted from the spores and fruiting bodies, have mitogenic activity [55,58].

Several enzymes have also been isolated from *G. lucidum*, such as cellulase, carboxyproteinase, amylase, endopectin methyltranseliminase, endopolygalacturonase, manganese superoxide dismutase, endo- and exo-polygalacturonase, isoforms of chymotrypsin inhibitors, and laccase isoenzymes [17].

It has also been found to contain nucleosides such as cystidine, adenosine, thymidine, guanosine, inosine, and uridine, as well as nucleotides such as guanine, adenine, hypoxanthine, thymine, and uracil [57].

Various vitamins, including vitamins B1, B2, B6, β-carotene, C, D, and E, have also been isolated from *G. lucidum* [59]. Additionally, a number of minerals have been isolated from *G. lucidum*, including sodium, potassium, calcium, phosphorus, magnesium, selenium, iron, carbon, zinc, copper, manganese, chromium, arsenic, silicon, aluminum, nickel, lead, cobalt, molybdenum, and fluoride [11,60]. Research suggests that *G. lucidum*’s polysaccharides and triterpenes are primarily responsible for its anti-tumor effects [61,62].

This review will focus on discussing the antitumor effects of *G. lucidum* secondary metabolites, particularly on hematologic malignancies like leukemia and lymphomas, explaining cellular patterns and molecular pathways through which compounds from this mushroom interact with neoplastic cells interfering with proliferative and survival signals and leading, finally, to apoptosis.

## 4. *Ganoderma lucidum* and Cancer

Cancer is the leading cause of death in developed countries and the second most common cause of death in developing countries, with one in six deaths being cancer-related [63]. Chemotherapy and radiotherapy are associated with various adverse events that can affect the nervous, pulmonary, skin, gastrointestinal, genitourinary, cardiovascular, hematological, and reproductive systems [64,65,66]. Because of these side effects, patients’ long-term adherence to treatment is often reduced and their quality of life is impaired [67]. Consequently, numerous oncological studies have been initiated in recent decades to search for new substances to be used as alternative anti-cancer drugs; for this purpose, phytochemicals represent important resources [68,69,70,71,72,73,74,75].

One of the most natural products widely studied in hematological malignancies like leukemia and lymphoma as a potential anticancer agent is *G. lucidum* [49]; in fact, recent in vitro and in vivo studies show promising results for its anticancer activity [27], since it is able to determine the activation of the host cell immune response [10], induction of cell differentiation, induction of phase II metabolizing enzymes, inhibition of urokinase-type plasminogen activator (uPA) and urokinase-type plasminogen activator receptor (uPAR) expression in tumor cells, direct cytotoxicity and inhibition of angiogenesis [30,76,77,78,79]. *G. lucidum* has been found to have an antiproliferative effect in both laboratory and animal experiments [80], a cytotoxic effect by interrupting the tumor cell cycle and inducing apoptosis [81,82,83], and also NK cell cytotoxicity against various tumor cell lines [84].

As mentioned earlier, the main metabolites with antitumor activity in this edible mushroom are polysaccharides and triterpenoids. Among polysaccharides, β-D-glucans show the main antitumor properties, especially β-1-3 and β-1-6-D-glucans [85,86], since they cause the activation of effector cells that bind leukocytes and serum-specific proteins, such as macrophages, T-helper cells, and natural killer cells [76]. Consequently, it increases the production of cytokines, e.g., interleukins, tumor necrosis factor α (TNFα), interferon (IFN), and nitric oxide (NO) [87]. The first evidence of the antitumor action of *G. lucidum* polysaccharides was observed in 180 cells from mouse sarcoma in 1981 [88]. Against cancer cells, ganoderic acids have significant pharmacological effects [89]. Ganoderic acid T (GA-T) was found to effectively inhibit metastasis in vivo and tumor cell invasion in vitro, reduce tumor cell proliferation, and exert cytotoxic effects on lung cancer cell line 95-D and human hepatoma SMMC-7721 cell lines [21,90,91]. Ganoderic acid DM (GA-DM) exhibits antiproliferative and antimetastatic properties on several human cancer cells, acting by modulating the androgen or estrogen receptor and inducing apoptosis [44,92,93]. By inhibiting metastasis and cell proliferation, ganoderic acid H (GA-H) and ganoderic acid A (GA-A) exhibit extraordinary effects on breast cancer [94]. Ganoderic acid Me (GA-Me) shows substantial tumor invasion capabilities, such as inhibiting the expression of metalloproteinase-9 (MMP9) and metalloproteinase-2 (MMP2) and inhibition of cell adhesion, as well as anti-metastatic outcomes [95,96,97].

## 5. Unravelling the Pathways and Mechanisms through which *Ganoderma lucidum* Acts

### 5.1. Induction of Apoptosis

After the exposure to the F3 polysaccharide fraction of *G. lucidum*, four hematopoietic cell lines were examined for apoptosis, and they showed to be able to induce the expression of IL-1, IL-6, IFN-gamma, TNF-alpha in monocytes–macrophages, and T lymphocytes, which, in turn, exert a proapoptotic activity [98,99,100,101,102,103]; moreover, these polysaccharides may also have immune-enhancing properties, perhaps as a consequence of their ability to stimulate cytokine production [104,105,106,107].

Among hematological malignancies, research focused on the anti-cancer effects of *G. lucidum* in cells from acute lymphoblastic leukemia, myeloid leukemia (HL-60, U937, K562, THP-1, NB4), non-Hodgkin’s lymphoma (NCEB-1, SUDHL6), and multiple myeloma (RPMI8226, ARH77, U266, NCI-H929); in detail, HL-60 myeloid leukemia cells exposed to 100 g/mL of F3 for 72 h became multinucleated and showed an increased DNA content with the result of 29% of G2/M arrest compared with the 12% of control cells. It is well known that apoptosis starts with a series of cascading events such as the depolarization of the mitochondrial membrane potential [72]; in fact, *G. lucidum* has the capability to alter the mitochondrial transmembrane electrical potential of HL-60 and U937 myeloid cells. When cells lose their electrical potential, the fluorescence of the kit used for experiments changes from red to green in a dose-dependent manner, indicating that apoptosis is in course [81].

A dynamic analysis of gene expression in THP-1 monocytic leukemia cells with *G. lucidum* treatment was conducted at various time points in order to determine whether F3 has similar interactions with death receptors that drive apoptotic pathways in leukemia cells. THP-1 cells experienced cell shrinkage, one of the signs of cell death, following a 48 h F3 treatment. Moreover, it was possible to observe nuclear chromatin condensation, which results in the deterioration of genomic DNA, as a marker of early-stage cell death; in fact, the proportion of chromatin-condensed cells significantly increased in the F3-treated culture [108].

Apoptosis pathways have been studied by scientists, who focused on six genes of the NF-κB signaling pathway, observing that they are up-regulated following F3 therapy, especially for those that concern MYD88, TRADD, interleukin 1 beta (IL1B), interleukin 1 alpha (IL1A), NFKBIA, and NFKB1. In detail, IL1B at low concentrations strongly elicited apoptotic responses as demonstrated by caspase-8 activation and DNA fragmentation [13]; soon after TNF engages TNFR1, NF-B is strongly activated, producing a pro-survival signal that must be suppressed in many cell types for TNF to induce apoptosis.

**Figure 1:**
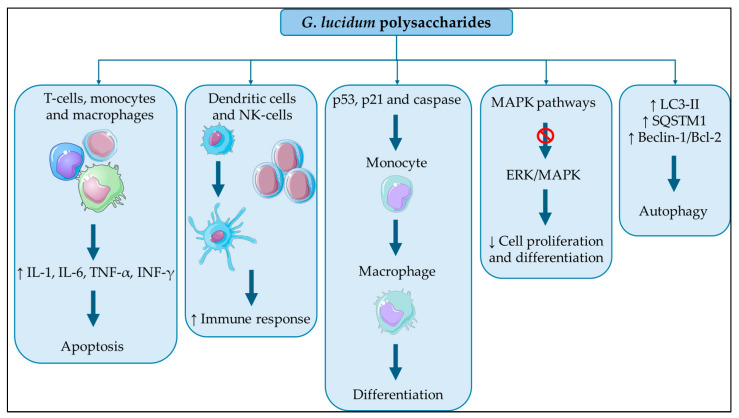
Mechanisms of action of Ganoderma lucidum in cancer. This figure was partly generated using Servier Medical Art, provided by Servier, licensed under a Creative Commons Attribution 3.0 Unported License.

All these findings lead us to state that, after F3 therapy, NF-KB signaling pathway activation leads to further THP-1 cell death [108] (Figure 1).

### 5.2. Stimulation of the Immune Response

The most effective antigen-presenting cells are called dendritic cells (DCs), and they have a special capacity to bridge innate and adaptive immunity; the present approach to examine the biology and differentiation of circulating DCs is mostly based on the differentiation of monocytes into circulating DCs using the cytokines GM-CSF and IL-4. Researchers have shown that *G. lucidum* polysaccharides can promote the maturation of human dendritic cells, boost monocytic leukemic cell proliferation, and trigger dendritic cell differentiation from monocytic leukemic blasts [109].

In leukemic cells from mice, polysaccharides from *G. lucidum* improve T-cell and B-cell surface markers and potentiate phagocytosis of macrophages and NK cell activity; in fact, NK cells help to get rid of altered tumor cells and are involved in non-specific antiviral and anticancer defense in human cancer cells [110,111]. Recent findings showed that NK cell activity was enhanced following treatment with *G. lucidum* extracts, while the percentages of Mac-3 and CD11b genes were reduced, and CD3 and CD19 were, instead, elevated, suggesting that F3 may increase the amount of T and B cells, respectively [112].

In a study involving animals, scientists discovered that mice given large doses of *G. lucidum* polysaccharides had considerably higher serum levels of IL-2, IL-6, IL-12, and TNF-a, and this fact could be explained by a control role of these molecules over the immune system in encouraging immune cells to secrete antitumor chemicals.

### 5.3. Macrophage-like Differentiation through Caspase and p53 Activation

The change in cell morphology from a monocytic phenotype to a macrophage-like phenotype and the increased cell adherence after receiving *G. lucidum* treatment for 24 and 48 h both supported the influence of F3 on cell differentiation; in fact, the decrease of nitroblue tetrazolium (NBT) and the evidence of cell cycle arrest in the G0/G1 phase indicate the impact of differentiation into cells that resemble macrophages. Moreover, the upregulation of CD11b, CD14, CD68, and MMP-9 and the downregulation of MPO strongly showed the influence of macrophage differentiation in THP-1 cells treated with F3.

Multiple experimental models have shown that wild-type p53 and p21 promote cellular differentiation of monocytes; in fact, when F3 is applied to THP-1 cells, p53 and p21 expressions are gradually increased during macrophage differentiation, and macrophage differentiation markers are decreased when p53 activity is inhibited. Additionally, the accumulation of cell cycle progression in the G0/G1 phase may be partially influenced by the actions of p53 and p21. According to these results, the p53-dependent mechanism controls cell differentiation and cell cycle arrest [113], and Mac-3 decreased, while macrophage phagocytosis increased because F3 stimulated the differentiation of monocytes into activated peritoneal macrophages [81,114,115,116,117,118].

One of the main pathways leading to apoptosis is the mitochondrial/caspase-mediated signaling cascade, and mitochondrial outer-membrane permeabilization (MOMP) plays a significant part in this route: cytochrome C is released into the cytoplasm due to MOMP collapse, which sets off the caspase cascade and apoptosis that follows. Researchers found that GT treatment increased the levels of procaspase-9, procaspase-3, cytochrome C, and PARP, which in turn caused apoptosis in K562 cells; furthermore, the production of FasL receptor, Fas protein, and caspase-8 regulates the death-receptor-mediated extrinsic pathway, which in turn promotes apoptosis. Following FasL activation, caspase-8 activation causes the proteolytic cleavage of mitochondrial-associated Bid to cause apoptotic cell death. Bid, a proapoptotic protein that is a substrate for caspase-8 targets the permeabilization of the mitochondrial membrane, linking the intrinsic and extrinsic apoptotic pathways.

In previous investigations, it was shown that *G. lucidum*-induced apoptosis was responsible for activating Fas, cleaving caspase-8 into an active form, and cleaving Bid in K562 cells, with the effects of a concentration-dependent treatment [119].

### 5.4. Activation of MAP-K Pathway

Since MAPK activation causes its downstream substrates to be successively phosphorylated, including transcription factors, protein kinases, and constitutive proteins, it is believed to be one of the key regulators of cell proliferation and differentiation; in fact, targeting MAPK signaling pathways has emerged as one of the key therapeutic approaches to halt tumor growth, in particular for concerning ERK, JNK (c-jun N-terminal kinase), and p38 MAPK. Therefore, using these kinases and the genes they regulate, the targeting effect of *G. lucidum* on MAPK pathways in leukemia cells has been studied [120]: ERK/MAPK signaling transduction is a traditional pathway that is also involved in the pathogenesis of leukemia, and a constitutive activation was found in some acute leukemic cells; this statement is supported by previous studies finding that untreated HL-60 cells both in vitro and in vivo had overexpression of p-ERK1/2.

It was hypothesized that *G. lucidum* may affect HL-60 cell proliferation by suppressing the ERK/MAPK pathway because it was found that when it was administered in high concentrations, the level of ERK1/2 phosphorylation was down-regulated [121]. Moreover, the expression of cyclin D1, an ERK-regulated downstream protein, is significantly downregulated, and, as a result, the G1/S phase transition that cyclin D1 controls is repressed. In addition to controlling cyclin D1 in acute leukemic cells, the ERK/MAPK pathway also works with members of the Bcl-2 proteins to prevent apoptosis; among them, the protein Bax can form a heterodimer that, in turn, controls cell apoptosis, but the ratio of Bcl-2 to Bax determines how sensitive it is to death signals. According to research, blocking ERK1/2 activity increased the activity of caspases such as caspase-3 while having no effect on the levels of the pro-apoptotic gene Bax or the production of the Bcl-2 protein: all these events lead to apoptosis.

Numerous cell genes, including c-myc and p53, participate not only in the advancement of the cell cycle but also in the control of cell apoptosis: in vitro studies showed increased levels of phosphorylated c-myc and p53 proteins after cells were treated with *G. lucidum*, suggesting that it was the cause of the c-myc- and p53-induced apoptosis [122]. The MAPK signaling pathways, which mediate significant physiological responses like proliferation, differentiation, and death are among the oldest signal transduction pathways and the most evolutionarily conserved signaling regulators. Though they are members of a vast MAPK family, JNK, p38 MAPK, and ERK are functionally distinct subgroups: the ERK cascade is regularly linked to pro-survival activity in a variety of cell types, while the JNK and p38 families seem to have proapoptotic effects; in detail, JNK promotes the transcription of c-Jun target genes by phosphorylating and activating c-Jun.

A number of cellular stressors, such as infection, hyperosmolarity, ultraviolet light, heat shock, and proinflammatory cytokines activate P38 MAPK, which acts at an early stage before mitochondrial failure and caspase activation; it has been demonstrated that p38 MAPK inhibition allows *G. lucidum* triterpenes to cause autophagy in colon cancer cells and alter ERK1/2 signaling to prevent breast cancer cells from behaving aggressively as a result of oxidative stress [123,124].

### 5.5. Induction of Cytoprotective Autophagy

In myeloid leukemia and solid tumors, a dynamic and self-catabolic process known as autophagy is involved in the breakdown and recycling of macromolecules and organelles. During these events, the activating enzymes Atg7 and Atg3 combine the cytosolic form of LC3-I with phosphatidylethanolamine to create LC3-II [125,126,127,128]. Another essential protein known as p62 or sequestosome 1 (SQSTM1) directly attaches to LC3-II through a particular sequence motif to produce autophagosomes, which then go through self-degradation during autophagy. Because it may interact with signaling proteins, the autophagy adaptor protein p62/SQSTM1 serves as a hub for signaling and represents an adaptive survival strategy in adult acute myeloid leukemia cells.

The results of previous investigations showed that *G. lucidum* treatment of K562 cells induced autophagy by increasing the accumulation of LC3-II and the expression of P62/SQSTM1; in fact, real-time protein-chain reaction (RT-PCR) results revealed an elevation of LC3 mRNA expression levels. In tumor suppression, autophagy and apoptosis-associated cell-death pathways are crucial and share some essential signals that influence cell survival or death; these two biological processes have the ability to inhibit one another.

It has been possible to identify the anticancer activities of substances obtained from natural sources and traditional Chinese medicine extracts using the pharmacological autophagy inhibitors 3-MA and CQ, which could block autophagy and increase caspase-3 cleavage, demonstrating that *G. lucidum*-mediated cytoprotective autophagy prevented apoptosis in K562 cells.

Beclin-1 is regarded as a key protein in the autophagy process, and its expression may be cytoprotective or encourage cell apoptosis; it is known to be inhibited under normal circumstances by Bcl-2 protein and separates from Bcl-2 in stress response, which causes autophagy to be triggered. In a time-dependent experiment, researchers found that incubation with *G. lucidum* significantly elevated Beclin-1/Bcl-2 expression levels, indicating the activation of autophagy in K562 cells [129,130]. Autophagy is strictly regulated by upstream modulators, namely, the PI3K/AKT/mTOR signaling pathway; natural bioactive substances stimulate autophagy via this mechanism in cancer cells; in fact, it was observed that blocking EFGR and the PI3K/AKT/mTOR signaling cascade may help cancer cells engage in autophagy: there is evidence of a significant reduction of the levels of EFGR, PI3K, phosphorylated PI3K, and AKT protein expression after F3 incubation [119] (Table 1 and Figure 1).

### 5.6. Ganoderma lucidum and Hematological Malignancies

The effects of *G. lucidum* on various hematological malignancies have been investigated largely (Table 2), and the antitumor effects of *G. lucidum* using a panel of 26 human cancer cell lines have been evaluated, including acute lymphoblastic leukemia (Blin-1, Nalm-6, Jurkat), myeloid leukemia (HL-60, U937, K562, THP-1, NB4), Burkitt’s lymphoma (Daudi, Ramos), non-Hodgkin’s lymphoma (NCEB-1, SUDHL6), multiple myeloma (RPMI8226, ARH77, U266, NCI-H929), prostate cancer (LNCaP, PC-3, DU145), colorectal cancer (HT-29), breast cancer (MCF-7, MDA-MB-231), non-small cell lung cancer (NCI-H520), and pancreatic cancer (PANCI, ASPC1, BxPC-3). The 26 cancer cell lines used were cultured for 96 h in the presence of 50 and 100 μg/mL *G. lucidum* extract, obtained by aqueous extraction from the fruiting bodies and enriched in ganoderic acid C2 (GA-C2), and their growth was evaluated. Subsequently, the six hematological cell lines (HL-60, U937, K562, Blin-1, Nalm-6, and RPMI8226) were more sensitive to GL and achieved a growth inhibition of 50%, so they were further studied. The effective dose to inhibit growth by 50% (ED50) was calculated for each cell line and ranged from 26 to 63 μg/mL. Cell cycle analysis was performed on HL-60, U937, RPMI8226, Nalm-6, and Blin-1 cells incubated for 72 h with or without *G. lucidum* extract at a concentration of 100 μg/mL. The results showed a G2/M arrest, especially in HL-60 cells (29% in treated vs. 12% in control cells). The increase in cells in the G2/M phase was minimal in RPMI8226 cells (20% in treated vs. 16% in control cells) and Nalm-6 cells (15% in treated vs. 12% in control cells), but there was no increase in the number of cells in the G2/M phase in Blin-1 or U937 cells. Apoptosis was then assessed by the Annessin V assay, and four cell lines (HL-60, U937, Blin-1, and RPMI8226) were treated with *G. lucidum* at concentrations of 50, 100, 150, and 200 μg/mL for 72 h. *G. lucidum* induced apoptosis in each of the cell lines in a dose-dependent manner, with the best results for HL-60 cells (approximately 92% apoptotic cells at the concentration of 200 μg/mL, after 72 h) and Blin-1 cells (approximately 42% apoptotic cells at the concentration of 200 μg/mL, after 72 h); U937 and RPMI8226 cells showed less apoptosis (32% and 21%, respectively, at the concentration of 200 μg/mL, after 72 h). The effect of *G. lucidum* on the expression of cell cycle and apoptosis-related proteins was investigated by Western blot analysis on U937 cells treated with increasing doses of *G. lucidum* extract (50, 100, 150, and 200 μg/mL) for 48 and/or 72 h, and an upregulation of p21 WAF1 and p27 KIP1 by *G. lucidum* extract was detected [81].

Another in vitro study, conducted by Calviño and colleagues, evaluated the effect of two aqueous extracts (E1, unboiled, obtained after centrifugation of fruiting bodies in sterile water at room temperature for 5 min; E2, boiled, obtained by resuspending the pellet in sterile water and boiling for 5 min) and a methanolic extract (E3, obtained after fruiting bodies were disrupted and resuspended in 10% ethanol; the solvent evaporated, and the compound was resuspended in dimethyl-sulfoxide) of *G. lucidum* on a murine model of interleukin-3 (IL-3)-modulated lymphoma, lymphoma DA-1 cells. A total of two cell lines were employed: DA-1 cells were kept at 37 °C and 5% CO_2_ in Iscove MDM medium with 10% fetal calf serum, 2 mm l-glutamine, and 2.5 × 10^−5^ m β-mercaptoethanol, and the WEHI-3B cell line was used as a source of conditioned medium that contained interleukin-3, which is essential for the survival of DA-1 cells. Cells were incubated with IL-3 and exposed to different concentrations of E1, E2, and E3 extracts or to 100 μm etoposide, which was used as a positive control for toxicity and induction of apoptosis, for 13, 19, and 24 h. Etoposide induced a decrease in cell viability with viability values of 70%, 58%, and 51% at 13, 19, and 24 h, respectively. Extracts E1 and E3 showed an effective reduction in cell viability of up to 70% and 36%, respectively, after 24 h of treatment. In contrast, extract E2 had no significant effect on viability. Treatment-induced apoptosis was then assessed and measured by quantifying subdiploid DNA. Extract E1 showed a similar level of time-dependent induction of cell death as a 100 μm etoposide. Extract E3 also showed significant induction of subdiploid DNA in DA-1 cells, although it was less than E1. In contrast, extract E2 showed very limited induction of cell death in DA-1 cells. Finally, Western blot analysis was used to assess the levels of certain proteins, as p53, Bax, Bcl-2, and Mdm-2 are involved in apoptosis processes after exposure to the different types of *G. lucidum* extract. In particular, an increase in p53 and a reduction in Mdm2 were observed in DA-1 cells treated with E1 and E3, but not in cells treated with E2, as was a reduction in the levels of Bcl-2 and NF-kB for all three extracts and activation of caspase 3 in cells exposed to E1 and E3 [131].

Extracts of *G. lucidum* E1, E2, and E3 were also used in another study conducted on a human leukemia cell line NB4. In this case, cells were treated with *G. lucidum* extracts E1, E2, E3, or GA-C2 for 19 h, using etoposide as a positive control. Treatment of NB4 cells with the E1 extract reduced cell viability to 77% compared to untreated control cells; the E2 extract also resulted in a reduction in cell viability, albeit less than E1; the E3 fraction showed a dose-dependent reduction in viability, up to a maximum value of 68%; in contrast, GA-C2 had no effect on cell viability. The induction of DNA fragmentation, an expression of apoptosis, was then investigated: extracts E1 and E3 produced significant DNA fragmentation, exceeding 40%; a lower effect was produced by treatment with GA-C2; while extract E2 did not result in significant DNA fragmentation. Furthermore, changes in some proteins involved in apoptosis were studied by Western blot in NB4 cells treated with E3 extract. The results showed a reduction in p53, Akt, Erk, NFκB, and Bcl-2 and an increase in Bax in treated cells compared to controls [132].

Wang and colleagues investigated the effect of active lipids from *G. lucidum* spores dissolved in ethanol on THP-1 cells, human acute monocytic leukemia cell lines, and HL-60 cells, human promyelocytic leukemia cells. The active lipids were obtained as follows: samples of *G. lucidum* spores were manually ground for four hours in a glass mortar, and then they were ultrasonically extracted using CH2Cl2 for two hours. After filtering the solution, the residue was extracted twice more using CH2Cl2. After combining the filtrates, the solvent was eliminated using a rotary evaporator. Finally, the lipids of *G. lucidum* spores were dissolved in ethanol. Cells were incubated with active lipids from *G. lucidum* spores at different concentrations (0, 0.25, 0.5, 1, and 2 mg/mL) for 48 h or at a concentration of 1 mg/mL for 0, 12, 24, 48, and 96 h. It was found that active lipids from *G. lucidum* spores induced the decrease in viability of THP-1 and HL-60 cells, as determined by trypan blue staining, in a dose- and time-dependent manner. Apoptosis was assessed by flow cytometry using FITC-conjugated Annexin V and PI in both cell lines, which showed high rates of apoptosis in a dose- and time-dependent manner after exposure to active spore lipids. Protein expression was then assessed by Western blot, and it was found that treatment with active lipids significantly decreased P-Akt production in THP-1 cells in a time-dependent manner, supporting the conclusion that apoptosis induced by active lipids in *G. lucidum* spores is mediated by downregulation of P-Akt; expression of P-ERK1/2 decreased and P-JNK1/2 increased in a time-dependent manner, whereas P-p38 MAPK expressions were not affected. Caspase-3, -8, and -9 activity were then measured in THP-1 cells incubated in the presence of active spore lipids, which showed a dose- and time-dependent up-regulation of these caspases, demonstrating that these caspases are involved in the apoptosis of THP-1 cells induced by active lipids of *G. lucidum* spores [124].

Other studies have been carried out on THP-1 cell lines, one of which evaluated the potential role in the induction of leukemic cell differentiation after exposure to a polysaccharide moiety isolated from the water-soluble residue of the *G. lucidum* polysaccharide, termed F3, which had been shown in a previous study to stimulate the inflammatory response through the expression of IL-1, IL-6, IL-12, and TNF-α [108]. F3 was obtained from the water-soluble residue of reishi and purified by gel filtration chromatography using a Sephacryl S-500 column with 0.1 N Tris buffer as an eluent. High-throughput microarrays were used to screen and study the dynamic patterns of gene expression, and the possible biological functions and physiological role of F3-treated leukemia cells were analyzed using a bioinformatics approach. THP-1 cells were treated with 30 μg/mL F3 and cell adhesion, a hallmark of macrophage differentiation, was examined by phase-contrast microscopy. F3 treatment resulted in the adhesion of approximately 45% of the cells; it significantly increased the reduction of NBT, a functional assay to assess the ability to produce superoxide during macrophage differentiation, and it also increased the population of THP-1 cells in the G0/G1 phase from 46 to 80% after treatment. Specific cellular markers and enzyme activities related to macrophage differentiation were analyzed, and overregulation of CD11b, CD14, CD68, and metalloproteinase-9 (MMP-9) and under-regulation of myeloperoxidase (MPO), a marker of the myeloid lineage, were noted. In addition, activation of p53 and caspase-3, -7, -8, and -9 was documented in THP-1 cells exposed to the F3 fraction. These data suggest that F3 polysaccharides from *G. lucidum* might have the potential to induce differentiation of leukemic cells into macrophage-like cells [113].

Zhong et al. evaluated the cytotoxicity of the 5th fraction of low-molecular-weight water-soluble polysaccharides extracted from the fruiting bodies of *G. lucidum* (GLP5) on the human acute T-cell leukemia cell line Jurkat. GLP5 was prepared as follows: 100 g of *G. lucidum* fruiting body powder was defatted by soaking in 500 mL of 95% ethanol. The defatted powder was dried and extracted with 300 mL of distilled water on a rotary shaker. The aqueous extract was centrifuged and the supernatant was dried under vacuum and washed with 200 mL of anhydrous ethanol, acetone, and ether to give the crude extracts. The crude polysaccharide was dissolved in 50 mL of distilled water and applied to a cellulose column equilibrated with distilled water and eluted with distilled water and various concentrations of NaCl solution. The eluates were concentrated and further fractionated by size exclusion chromatography. Jurkat cells and the immortalized human epidermal HaCat cell line were used for the experiments; the cells were exposed to different concentrations of GLP (0 to 1000 mg/L) for 48 h. Cell viability was assessed by MTT assay, Jurkat cell viability decreased after GLP5 exposure in a concentration-dependent manner from more than 95% at 0 mg/l to less than 5% at 600 mg/L; changes in HaCat cell viability were not significant. Then, apoptosis in HaCat and Jurkat cells after exposure to 25 and 50 mg/l GLP5 was assessed using flow cytometry. The apoptosis rate increased from 0.92% in untreated cells to 4.35% and 49.22% after exposure to 25 and 50 mg/L GLP5, indicating that GLP5 could cause Jurkat cell death through apoptosis. On the other hand, the apoptosis rate in HaCat cells remained unchanged. Furthermore, in Jurkat cells after exposure to GPL5 (25 mg/L and 500 mg/L) by Western blot, the following were assessed: caspase-3 expression, which was significantly upregulated; Bcl2 expression, which was significantly reduced; and Bax expression, which was significantly increased [133].

An in vivo study was performed on 50 BALB/c mice injected with WEHI-3 leukemic cells to assess whether *G. lucidum* promoted immune responses. The mice were randomly divided into five groups: groups I and II were the control; group III was injected with WEHI-3 cells; group IV was injected with WEHI-3 cells and then treated with 3 mg/kg of *G. lucidum* administered via a gastric tube; and group V was injected with WEHI-3 cells and then treated with 6 mg/kg of *G. lucidum* administered via gastric tube. The mice were treated daily for 2 weeks. Groups I and II had 100% survival. Group III had the lowest survival rate (30%). Group IV had a higher survival rate than group III. Group V had the highest survival rate compared with groups III and IV. The groups treated with *G. lucidum* had a reduction in splenic weight compared with controls. Blood samples were taken from each mouse and analyzed by flow cytometry. Treatment with *G. lucidum* increased CD3 and CD19 levels but reduced Mac-3 levels and had little or no effect on CD11b, suggesting that T and B cell precursor differentiation was promoted but macrophages were inhibited. *G. lucidum* also promoted phagocytosis by macrophages of peripheral blood mononuclear cells (PBMCs) and promoted natural killer cell activity. These data suggest that *G. lucidum* affects murine leukemia WEHI-3 cells in vivo [112].

## 6. Conclusions

Natural product libraries offer a remarkable collection of new compounds that have the potential to serve as building blocks for the development of ground-breaking medicines [133].

*Ganoderma lucidum* grows on oak and plum trees and occurs in different colors and shapes, containing glycoproteins, amino acids, enzymes, vitamins, polysaccharides, triterpenoids, meroterpenoids, sesquiterpenoids, steroids, alkaloids, benzopyran derivatives, and benzoic acid derivatives [48,49,50]. Due to their pharmacological properties, polysaccharides and triterpenoids are the main groups of chemicals demonstrated to be active in various diseases like cancer.

Among neoplasms, hematological malignancies have high rates of morbidity and mortality and recognize different risk factors such as smoking, obesity, excessive consumption of alcoholic drinks, and an incorrect diet, but unmodifiable factor risks also exist and they depend on individual genetic traits [35]. The treatment for patients does not include only a rise in lifetime but also an improvement of quality of life; in this context, molecules from the vegetal world like *G. lucidum* are shown to be able to exert anti-cancer effects by targeting different pathways like that related to apoptosis or enhancing the immune system against neoplastic cells [134].

Moreover, oxidative stress leads to genetic modifications in nucleic acids, preventing programmed cell death, and it is certainly associated with an elevated risk of carcinogenesis [36,135]. The polysaccharidic fraction of this mushroom has shown the capability to induce apoptosis in cells from acute myeloid leukemia and other hematological neoplasms interacting with the pro-apoptotic NF-KB pathway and causing the depolarization of mitochondrial membrane potential. Furthermore, the immune system reacts in a positive way to *G. lucidum* administration because, as demonstrated in leukemic cells from mice, F3 improves T-cell and B-cell surface markers and potentiates phagocytosis of macrophages and NK cell activity [72,110]. The research focused also on the capacity of *G. lucidum* polysaccharides to determine caspase and p53 activation and, in turn, the macrophage-like differentiation of monocytes and a stimulation of cytoprotective autophagy [12,25].

Currently, G. lucidum has no clinical applications, but it could be very useful if the evidence we presented would encourage the synthesis of new drugs capable of fighting neoplastic cells of leukemia, lymphoma, and multiple myeloma. It is needed, however, to be borne in mind that the active substances contained in *G. lucidum* have a biological action on the human organism and may, therefore, be burdened with undesirable effects or interact with synthetic drugs [86]. Teratogenic and toxic effects of Ganoderma lucidum were reported on zebrafish embryos [136], and an anti-platelet effect was also exhibited by an extract of *G. lucidum*. Furthermore, it also revealed cytotoxicity in platelets as well as leukocytes, as shown by an increase in LDH leakage [137]. Further in vivo studies are required to use these substances safely in clinical practice.

## Figures and Tables

**Table 1:** Anticancer mechanisms of action of *Ganoderma lucidum* in cancer and related involved molecules.

Mechanism	Involved Molecules	References
Induction of apoptosis	Apo2L, DR4, DR5, DcR1, DcR2	[14]
Stimulation of the immune response	Dendritic cells, NK cells	[18]
Macrophage-like differentiation through caspase and p53 activation	Caspase-8, PARP, FasL, p53, p21	[12]
Activation of MAP-K pathway	ERK, c-jun N-terminal kinase and p38	[29]
Induction of cytoprotective autophagy	Sequestosome 1	[25]

**Table 2:** Summary of studies on hematological malignancies and *Ganoderma lucidum*.

Tested Substances	Object of the Study	Main Outcomes	References
Aqueous extract enriched with GA-C2	In vitro 26 human cancer cell lines: Blin-1, Nalm-6, Jurkat, HL-60, U937, K562, THP-1, NB4, Daudi, Ramos, NCEB-1, SUDHL6, RPMI8226, ARH77, U266, NCI-H929, LNCaP, PC-3, DU145, HT-29, MCF-7, MDA-MB-231, NCI-H520, PANCI, ASPC1, BxPC-3	The aqueous extract resulted in growth inhibition of cell lines HL-60, U937, K562, Blin-1, Nalm-6, RPMI8226. G2/M arrest was mainly observed in HL-60 cells. It induced apoptosis in a dose-dependent manner in the HL-60, U937, Blin-1 and RPMI8226 cell lines. U937 cells treated with aqueous extract showed ↑ of p21 WAF1 and p27 KIP1.	[81]
Unboiled aqueous extract E1, boiled aqueous extract E2, methanolic extract E3	In vitro Mouse lymphoma DA-1 cells	The extract demonstrated the ability to ↓ cell viability in DA-1 cells, induced DNA fragmentation and determined changes in protein expression of apoptosis factors.	[131]
Unboiled aqueous extract E1, boiled aqueous extract E2, methanolic extract E3, GA-C2	In vitro leukemia cell line NB4	The extract demonstrated the ability to reduce cell viability in NB4 cells, induced DNA fragmentation and determined changes in protein expression of apoptosis factors.	[132]
Active lipids from spores dissolved in ethanol	In vitro human acute monocytic leukemia cell lines, THP-1, and human promyelocytic leukemia cells, HL-60	Active lipids from spores induced ↓ viability and ↑ of apoptosis in a dose- and time-dependent manner in THP-1 and HL-60 cells. ↓ expression of P-Akt and P-ERK1/2, ↑ P-JNK1/2, and ↑ of caspases-3, -8, and -9 were demonstrated in THP-1 cells.	[124]
Polysaccharide moiety isolated from the water-soluble residue of the polysaccharide F3	In vitro human acute monocytic leukemia cell lines, THP-1	The F3 fraction ↑ cell adhesion in THP-1 cells, ↑ reduction of NBT, G0/G1 phase cell cycle arrest, ↑ of CD11b, CD14, CD68 and MMP-9, and ↓ of MPO, activation of p53 and caspases -3, -7, -8 and -9.	[113]
5th fraction of low-molecular-weight water-soluble polysaccharides extracted from fruiting bodies (GLP5)	In vitro human acute T-cell leukemia cell line Jurkat	GPL5 resulted in ↓ viability and ↑ apoptosis rates in Jurkat cells and not in HaCat cells. Furthermore, in Jurkat cells there was ↑ of caspase-3 and Bax hypo-expression of Bcl2.	[133]
The fungus in its entirety	In vivo BALB/c mice injected with WEHI-3 leukemia cells	It ↑ the survival rate in BALB/c mice injected with WEHI-3 leukaemic cells; ↓ spleen weight; increased CD3 and CD19 levels; promoted phagocytosis by macrophages of (PBMCs); and promoted natural killer cell activity.	[112]

## Abbreviations

DCs, dendritic cells; FYGL, fudan-yueyang-*G. lucidum*; GA-A, ganoderic acid A; GA-C2, ganoderic acid C2; GA-DM, ganoderic acid DM; GA-H, ganoderic acid H; GA-Me, ganoderic acid Me; GA-T, ganoderic acid T; IFN, interferon; IL1A, interleukin 1 alpha; IL1B, interleukin 1 beta; IL-3, interleukin-3; JNK, c-jun N-terminal kinase; MMP2, metalloproteinase-2; MMP-9, metalloproteinase-9; MOMP, mitochondrial outer-membrane permeabilization; MPO, myeloperoxidase; NBT, nitroblue tetrazolium; NO, nitric oxide; PBMCs, peripheral blood mononuclear cells; RT-PCR, real-time protein-chain reaction; SQSTM1, sequestosome 1; TNFα, tumor necrosis factor α; uPA, urokinase-type plasminogen activator; uPAR, urokinase-type plasminogen activator receptor.
